# Foliar-Applied Glutathione Mitigates Cadmium-Induced Oxidative Stress by Modulating Antioxidant-Scavenging, Redox-Regulating, and Hormone-Balancing Systems in *Brassica napus*

**DOI:** 10.3389/fpls.2021.700413

**Published:** 2021-09-13

**Authors:** Ha-il Jung, Tae-Gu Lee, Jinwook Lee, Mi-Jin Chae, Eun-Jin Lee, Myung-Sook Kim, Goo-Bok Jung, Amoakwah Emmanuel, Sangho Jeon, Bok-Rye Lee

**Affiliations:** ^1^Division of Soil and Fertilizer, National Institute of Agricultural Sciences, Rural Development Administration, Wanju, South Korea; ^2^Department of Plant Science and Technology, Chung-Ang University, Anseong, South Korea; ^3^Crop Cultivation and Environment Research Division, National Institute of Crop Science, Rural Development Administration, Suwon, South Korea; ^4^Division of Climate Change and Agroecology, National Institute of Agricultural Sciences, Rural Development Administration, Wanju, South Korea; ^5^Council for Scientific and Industrial Research-Soil Research Institute, Academy Post Office, Kwadaso, Ghana; ^6^Asian Pear Research Institute, Chonnam National University, Gwangju, South Korea

**Keywords:** ascorbate-glutathione-NADPH cycle, cadmium phytotoxicity, glutathione, hormone, oilseed rape

## Abstract

The antioxidant glutathione (GSH) mitigates adverse physio-metabolic effects and defends against abiotic types of stress, such as cadmium (Cd) stress. However, its function and role in resisting Cd phytotoxicity by leveraging plant antioxidant-scavenging, redox-regulating, and hormone-balancing systems have not been comprehensively and systematically demonstrated in the Cd-hyperaccumulating plant *Brassica napus* L. cv. Tammi (oilseed rape). In this study, the effects of exogenously applied GSH to the leaves of *B. napus* seedlings exposed to Cd (10 μM) were investigated. As a result, Cd stress alone significantly inhibited growth and increased the levels of reactive oxygen species (ROS) and the bioaccumulation of Cd in the seedlings compared with those in unstressed controls. Furthermore, Cd stress induced an imbalance in plant stress hormone levels and decreases in endogenous GSH levels and GSH redox ratios, which were correlated with reductions in ascorbate (AsA) and/or nicotinamide adenine dinucleotide phosphate (NADPH) redox states. However, the exogenous application of GSH to Cd-stressed *B. napus* seedlings reduced Cd-induced ROS levels and enhanced antioxidant-scavenging defenses and redox regulation by both increasing seedling AsA, GSH, and NADPH concentrations and rebalancing stress hormones, thereby enhancing Cd uptake and accumulation. These results demonstrate that GSH improved plant redox status by upregulating the AsA-GSH-NADPH cycle and reestablishing normal hormonal balance. This indicates that exogenously applied GSH can mitigate Cd phytotoxicity in *B. napus* and possibly other plants. Therefore, GSH can potentially be applied to Cd-polluted soil for plant remediation.

## Introduction

Cadmium (Cd), a highly toxic heavy metal, is a non-essential element for all organisms. Despite its toxicity to human health and in agricultural production, the levels of Cd in potential agricultural soils and water bodies are increasing, primarily because of agricultural, urban, and industrial activities (Reeves and Chaney, 2008; Nakamura et al., 2013). Furthermore, Cd is one of the most hazardous materials to human health. Since humans are on the top of the food chain, they are especially affected by the bioaccumulation and biomagnification of Cd. Even low Cd concentrations can negatively affect numerous metabolic processes by replacing essential metal ions in both humans and plants that, in turn, disturbs the activities of essential enzymes (Farooq et al., 2018; Jung et al., 2018).

The mobility of cadmium in soil and water is very high, which makes it possible for the metal to be easily translocated and accumulated by plants. This subsequently leads to the inhibition of normal plant growth, which then causes oxidative stress through the production of reactive oxygen species (ROS), such as hydrogen peroxide (H_2_O_2_) and superoxide anion radicals (${\text{O}}_{2}^{•\text{–}}$). The overproduction of ROS caused by excessive Cd accumulation not only destroys cell membranes through lipid peroxidation, but also impairs the biosynthesis of essential metabolites required for normal plant growth. This phenomenon severely disrupts normal vital plant functions, such as photosynthesis, respiration, ion uptake, redox homeostasis, and hormone balance (Singh S. et al., 2016).

Plants use both non-enzymatic and enzymatic antioxidant scavenging pathways to decrease cadmium-induced oxidative stress. The non-enzymatic antioxidant defense pathway, in particular, uses antioxidant or redox metabolites containing ascorbic acid (AsA), glutathione (GSH), and NADPH to reduce the excessive accumulation of ROS. The enzymatic defense pathway for oxidative stress responses, on the other hand, involves the use of antioxidant and redox enzymes, especially superoxide dismutase (SOD), catalase (CAT), ascorbate peroxidase (APX), monodehydroascorbate reductase (MDHAR), dehydroascorbate reductase (DHAR), and glutathione reductase (GR) (Shekhawat et al., 2010; Hasanuzzaman et al., 2017).

Glutathione, which primarily functions as a redox buffer and/or antioxidant, is biosynthesized in the cytosol and the chloroplast stroma of plant cells (Rauser, 1995; Nakamura et al., 2013). Moreover, it is a precursor of phytochelatins (PCs), which sequester toxic heavy metals, such as Cd, in the vacuole (Salt and Rauser, 1995; Sooksa-Nguan et al., 2009). To date, the effectiveness of GSH in reducing oxidative stress caused by heavy metals has been documented for various plants grown in soil or hydroponic solutions treated with GSH (Shri et al., 2009; Chen et al., 2010; Noctor et al., 2012; Mostofa et al., 2014; Jung et al., 2019). However, the practical application of treating soil with GSH is likely to incur high treatment costs, especially when one considers that soil microbial activity, soil moisture content (which influences soil oxygen availability and, therefore, soil redox reactions), and soil pH may interfere with the absorption of GSH by plants, reducing the overall effectiveness of soil GSH treatments (Kim et al., 2017; Jung et al., 2019; Hussain et al., 2021). To overcome these practical barriers, Jung et al. (2019) affirmed in a previous experiment that the foliar application of GSH on arsenic (As)-exposed rice is effective in reducing the phytotoxicity of As in monocotyledonous rice plants.

Hydroponic glutathione treatments have been used to alleviate increases in reactive oxygen species that are known to be induced by environmental forms of stress, such as drought, salinity, high and low temperatures, and heavy metal stress (Noctor et al., 2012; Jung et al., 2019). Furthermore, Chen et al. (2010) and Shri et al. (2009) reported that a hydroponic treatment with GSH was effective in reducing Cd and As toxicity in barley and rice seedlings, respectively. Mostofa et al. (2014) opined that foliar applications of GSH increased the resistance of copper (Cu) toxicity and reduced accumulation of Cu. Jung et al. (2019) demonstrated that GSH mitigates As-induced oxidative stress and enhances As translocation into shoots by maintaining the homeostasis of the AsA-GSH cycle in rice plants. Interestingly, Nakamura et al. (2019) recently reported that foliar applications of GSH to oilseed rape seedlings increased the root-to-shoot translocation of zinc (Zn), which is an essential metal ion for maintaining the homeostasis of important metabolic processes.

Plant hormones are also involved in activities that decrease the adverse influences of various abiotic stress factors, such as Cd toxicity (Singh and Prasad, 2014; Xia et al., 2015). By disturbing the homeostasis of critical hormones, Cd comprehensively disturbs plant physiological, biochemical, and metabolic responses and inhibits ordinary plant growth and development (Agami and Mohamed, 2013; Asgher et al., 2015; Karam et al., 2017). However, the plant hormones disturbed by Cd are also implicated in other plant stress responses, such as oxidative stress responses. In particular, as a stress-associated plant hormone, abscisic acid (ABA) is a critical regulator of plant growth, development, and environmental stress responses (Xiong et al., 2002; Horn et al., 2013). Jasmonic acid (JA), on the other hand, regulates plant growth, development, and abiotic and biotic stress responses (Litwack, 2005; Piotrowska et al., 2009; Kazan and Manners, 2012). In addition, salicylic acid (SA) is a signal factor in plant defenses against environmental stress factors, such as salinity (Khan et al., 2014), drought (Kang et al., 2012), heavy metals (Zhang et al., 2011), and pathogens (Qi et al., 2018). The functions and roles of these plant hormones, ABA, JA, and SA, in mitigating oxidative stress induced by heavy metal stress have been reported in numerous plants (Piotrowska et al., 2009; Zhang et al., 2011; Asgher et al., 2015; Kaya et al., 2020). Moreover, Asgher et al. (2015) reported a comprehensive and systematic review of the critical role of these hormones in the oxidative stress responses of numerous plant species subjected to Cd stress. Consequently, elucidating the effects of GSH on hormone homeostasis in plants exposed to Cd toxicity should not only provide invaluable insights into the impact of the antioxidant on plant hormonal balance, but also inform us of the ability of GSH to mitigate the disruptive effects of Cd on hormone homeostasis.

Oilseed rape (*Brassica napus* L.) plants have great potential as cadmium hyperaccumulators due to their strong cadmium uptake and high cadmium accumulation ability, high cadmium tolerance with low growth inhibition, fast plant growth and development rates, and larger biomass compared with that of other natural heavy metal accumulators (Meng et al., 2009; Cojocaru et al., 2016; Hasanuzzaman et al., 2017; Chen et al., 2018; Jung et al., 2020; Zhang et al., 2020). Because of these advantages, oilseed rape plants are candidates for the eco-friendly phytoextraction of Cd (Ahmad et al., 2015; Hasanuzzaman et al., 2017). Therefore, we examined a new approach using foliar applications of GSH to elucidate the overall mechanisms by which Cd toxicity in oilseed rape plants may be mitigated through an evaluation of the effects of GSH on plant ROS and antioxidants, redox, and hormone homeostasis. The overall goal was to determine whether foliar applications of GSH can be practically deployed to remediate Cd-polluted soil using plants, specifically oilseed rape.

## Materials and Methods

### Plant Material and Stress Treatment

Oilseed rape (*B. napus* L. cv. Tammi) seeds were prepared under sterilized conditions based on a previous protocol (Jung et al., 2020). The seeds were covered with two paper towel layers that were moistened with distilled water at a temperature of 25°C for 48 h. This provided conductively moist conditions for effective seed germination. To ensure the uniformity of the plants, the germinated seeds were transferred into a plastic container (0.46 × 0.32 × 0.17 m, 0.147 m^2^ surface area) filled with a perlite-containing hydroponic solution for cultivation. The uniform three-leaf-stage seedlings were then transferred into plastic growing containers (two plants per container) for growing the plant material.

The plastic growing containers (0.30 × 0.25 × 0.15 m, 0.075 m^2^ surface area) were covered with a hydroponic solution containing macroelements and microelements in the following concentrations: 1.5 mM NH_4_NO_3_, 0.045 mM K_2_HPO_4_, 0.505 mM KH_2_PO_4_, 1 mM K_2_SO_4_, 3 mM CaCl_2_, 0.5 mM MgSO_4_, 0.4 mM Fe-EDTA, 14 μM H_3_BO_3_, 5 μM MnCl_2_·4H_2_O, 3 μM ZnSO_4_·7H_2_O, 0.7 μM CuSO_4_·5H_2_O, 0.1 μM Na_2_MoO_4_·2H_2_O, and 0.1 μM CoCl_2_·6H_2_O. The pH of the hydroponic solution was adjusted to 5.6, and the solution was continuously aerated and refilled every 5 days (Lee et al., 2016). Plants were grown in the hydroponic solution in a greenhouse at the National Institute of Agricultural Sciences, RDA, Wanju, Republic of Korea, under natural sunlight conditions at 27/22°C and 60/80% relative humidity day/night conditions, respectively. Furthermore, to minimize environmental variation, the plastic containers were rearranged every day during the plant growth and treatment period based on the experimental layout of a randomized complete block design with three replicates per treatment. Five-leaf seedlings were then treated with 10 μM of Cd (CdCl_2_) *via* the hydroponic solution. Then, 50 (162.7 μM) and 100 mg kg^−1^ (325.4 μM) of foliar GSH treatments and 2-ml L^−1^ of a commercial surfactant (20% sodium lignosulfonate and 10% polyoxyethylene alkyl aryl ether) were simultaneously applied (Jung et al., 2019, 2020).

Based on the results of a previous study by Jung et al. (2019), we knew that foliar glutathione alone did not affect the phenotypic characteristics of *B. napus* plants compared with untreated controls. Therefore, in this study, we did not examine GSH treatments alone, except for when we were testing the response of plant growth characteristics. In this study, GSH was applied once using a hand-held sprayer containing a 1,000-L ha^−1^ solution through flat-fan spray tips (Jung et al., 2019). Then, 10 days after the application of the treatment, liquid nitrogen was used to freeze the fresh root and leaf samples, after which, the samples were stored at −80°C until further analyses of the ROS, redox, and hormone levels.

### Plant Growth Characteristics

Shoot fresh weight (FW), shoot dry weight (DW), root dry weight, and shoot water content (WC) were evaluated to determine the effect of glutathione application on oilseed rape seedlings in cadmium-treated hydroponics with or without glutathione treatment based on a previous protocol (Jung et al., 2019, 2020). To do this, the seedlings were separated into roots and shoots 10 days post-treatment, and shoot FW was determined afterward. The shoots were then excised for the measurement of shoot WC, and their FW was immediately weighed. Finally, the dry weight was measured after the samples were dried in an oven at 60°C for 72 h.

### Analysis of Cd Concentration and Accumulation

Cadmium concentration and accumulation were determined based on the methods proposed by Jung et al. (2019, 2020). The *B. napus* root and shoot samples were thoroughly washed with tap water and then re-washed with deionized water for ~5 min. Afterward, the washed root and shoot organs were oven-dried at 60°C for 72 h and then powdered. To measure the Cd concentration in the roots and shoots of the oilseed rape plants, the powdered samples (200 mg) were digested using a Graphite Block Acid Digestion System (ODLAB Co., Ltd., Seoul, Republic of Korea). The digested solutions were then cooled to ambient temperature after digestion, diluted to 100 ml with ultrapure water, and filtered through Grade No. 40 filter papers (Whatman Co., Buckinghamshire, United Kingdom). Cd concentration was measured using an inductively coupled plasma-mass spectroscopy (ICP-MS) system (Agilent 7900; Agilent Technologies Inc., Santa Clara, CA, United States) and calculated as follows: Cd accumulation (μg plant^−1^) = Ca × Cb, where Ca is the Cd concentration and Cb is the DW in the roots and shoots of an oilseed rape plant, respectively.

### Estimation of ROS (${\text{O}}_{2}^{•\text{–}}$ and H_2_O_2_) and Lipid Peroxidation

The determination of reactive oxygen species, namely, superoxide anion radical (${\text{O}}_{2}^{•-}$) and hydrogen peroxide (H_2_O_2_), was performed according to the previous protocols developed by Elstner and Heupel (1976) and Jana and Choudhuri (1982), respectively. Superoxide anion radical concentration was measured by hydroxylamine oxidation. To do this, 1 ml of a 50-mM KPO_4_ buffer (pH 7.8) was added to 50 mg of freeze-dried and powdered root and leaf organs and centrifuged at 12,000 × *g* for 10 min at 4°C. Then, 0.5 ml of the supernatant was mixed with 1 ml of 10 mM NH_2_OH-HCl and incubated for 1 h at 25°C. Next, the mixture was treated with 1 ml of 7 mM *N*-(1-Naphthyl) ethylenediamine and 17 mM *p*-aminobenzene sulfonic acid for 20 min at room temperature. The absorbance of the mixture was read at 530 nm. A standard NaNO_2_ curve was used for calculations. For H_2_O_2_ determination, the roots and leaves of the samples were extracted with a phosphate buffer (pH 6.8) and treated with titanium chloride. The absorbance was immediately read at 410 nm and calculated using a coefficient of absorbance of 0.28 μM^−1^ cm^−1^. In addition, with regard to lipid peroxidation, malondialdehyde (MDA) production was analyzed using the thiobarbituric acid method developed by Buege and Aust (1978). A sample absorbance at 532 nm was measured and corrected for non-specific turbidity by subtracting this value from the absorbance value at 600 nm. Malondialdehyde concentration was calculated using an extinction coefficient of 156 mM^−1^ cm^−1^.

### Assessment of AsA/DHA, GSH/GSSG, and NADPH/NADP^+^

Ascorbate (reduced form) and dehydroascorbate (DHA, oxidized form) concentrations and glutathione (reduced form) and GSH disulfide (GSSG, oxidized form) concentrations were determined according to protocols outlined in Jung et al. (2019). The reduced and total AsA (AsA + DHA) levels were determined using a standard curve of AsA after reading the absorbance at 525 nm. The difference between the total AsA concentration and the reduced ascorbate concentration was then used as the oxidized DHA concentration.

Glutathione concentration was determined by a microplate assay using GSH/GSSG Kit GT 40 (Oxford Biomedical Research Inc., Rochester Hills, MI, United States). The absorbance of each sample was monitored at 412 nm for 1 min, and the oxidized GSSG and total GSH (GSH + GSSG) levels were calculated using the standard curves of GSSG and GSH, respectively. The recalculation of the reduced GSH concentration was then performed by finding the difference between the total GSH and GSSG concentrations.

The levels of nicotinamide adenine dinucleotide phosphate (reduced form) and nicotinamide adenine dinucleotide^+^ (oxidized form) were assessed using the method proposed by Queval and Noctor (2007). For NADPH and NADP^+^ extraction, frozen root and leaf organs were ground in liquid nitrogen and then re-suspended with 0.8 ml of 0.2 M HCl and 0.2 M NaOH, respectively. A 100-μl aliquot of each extract was heated at 95°C for 1 min and cooled in an ice bath. The supernatant was neutralized with 0.2 M of HCl to a final pH of 7–8 for the NADPH assay and counteracted with 0.2 M NaOH to a final pH of 5–6 for the NADP^+^ assay. Forty microliters were added to each reaction mixture containing 0.1 M hydroxyethyl piperazine ethane sulfonicacid (HEPES) (pH 7.5) that consisted of 2 mM disodium ethylenediaminetetraacetate dihydrate (Na_2_EDTA), 1.2 mM dichlorophenolindophenol (DCPIP), 20 mM phenazine methosulfate (PMS), and 10 mM glucose-6-phosphate. The reaction was then initiated by adding 2 μl of glucose 6-phosphate dehydrogenase (G6PDH, 200U). The NADPH and NADP^+^ concentrations were determined using a curve with standards of 1–100 pmol.

### Enzyme Activity Assays of the Antioxidant-Scavenging and Redox-Regulating Systems

To measure enzyme activity, the oilseed rape plant root and leaf samples were homogenized with a 100-mM potassium phosphate buffer, pH 7.5, containing 1% polyvinylpyrrolidone-40, 1 mM of phenylmethylsulfonyl fluoride, and 2 mM of ethylenediaminetetraacetic acid (EDTA) (Lee et al., 2013). After enzyme extraction, protein concentration was assessed according to the method of Kruger (1994). Based on previously published methods (Chen and Asada, 1989; Lee et al., 2009, 2013), SOD (EC 1.15.1.1), CAT (EC 1.11.1.6), and APX (EC 1.11.1.11) activities were assayed. Briefly, SOD activity was determined by measuring the ability of SOD to inhibit the photoreduction of nitrobluetetrazolium (NBT), in which 1 unit of enzyme activity was defined as the amount of enzyme required to inhibit 50% of NBT photoreduction, in comparison with the enzyme amount in tubes lacking the plant extract. Similarly, CAT activity was determined by measuring the decrease in absorbance at 240 nm and then recorded as an indicator of H_2_O_2_ degradation. It was calculated using an extinction coefficient of 36 mM^−1^ cm^−1^. Furthermore, APX activity was determined by measuring the decrease in absorbance at 290 nm using an extinction coefficient of 2.8 mM^−1^ cm^−1^. Monodehydroascorbate reductase (EC 1.6.5.4) and dehydroascorbate reductase (EC 2.5.1.1.8) activities were assayed using the methods presented by Nakano and Asada (1981) and Hossain et al. (1984), respectively. Monodehydroascorbate reductase activity was evaluated by monitoring the decrease in absorbance at 340 nm for 1 min as NADPH was oxidized and then calculated using an extinction coefficient of 6.2 mM^−1^ cm^−1^. Dehydroascorbate reductase activity, on the other hand, was measured by monitoring the increase in absorbance at 265 nm for 1 min as DHAR was reduced and then calculated using an extinction coefficient of 14 mM^−1^ cm^−1^. The activity of GR (EC 1.6.4.2) was also assayed following the detailed procedure of Rao et al. (1996), which involved monitoring the decrease in absorbance at 340 nm for 1 min as NADPH was oxidized, and also calculated using an extinction coefficient of 6.2 mM^−1^ cm^−1^.

### Hormone Analysis

Abscisic acid, jasmonic acid, and salicylic acid levels were measured in the roots and leaves of the oilseed rape plants according to the methods described by Pan et al. (2010). In brief, 50 mg of the sample was extracted using 500 μl of the extraction solvent 2-propanol/H_2_O/concentrated HCl (2:1:0.002, v/v/v) containing d_6_-ABA, H_2_Jas, and d_6_-SA, internal standards for ABA, JA, and SA, respectively, for 24 h at 4°C. Dichloromethane (1 ml) was added to the supernatant, and the mixture was shaken for 30 min at 4°C. This mixture was then centrifuged at 13,000 × *g* for 5 min at 4°C. After centrifugation, the solvent from the lower phase was poured into a clean screw-cap glass vial, dried under nitrogen conditions, and dissolved in pure methanol. The completely dissolved extract, ensured by vortexing and sonication, was filtered through a 0.22-μm organic membrane and then transferred to a reduced volume liquid chromatography vial. Hormone levels were subsequently analyzed using a reverse phase C18 Gemini high-performance liquid chromatography (HPLC) column for an HPLC–electrospray ionization tandem mass spectrometry (ESI–MS/MS) analysis. Agilent 1100 HPLC (Agilent Technologies Inc., Santa Clara, CA, United States), Waters C_18_ (Waters Corp. Milford, MA, United States) column (150 × 2.1 mm, 5 μm), and API3000 MSMRM (Applied Biosystems, Foster City, CA, United States) were used for the analysis.

### Statistical Analyses

The experimental design of this study was a randomized complete block design with three replicates per treatment. All the data obtained were analyzed using statistical analysis software (SAS ver. 9.2; SAS Institute Inc., Cary, NC, United States). An ANOVA was applied to evaluate the effects of the treatment on plant growth characteristics, Cd concentration and accumulation, ROS, the antioxidant scavenging system, and plant hormones based on organ (leaf and root) differences. The mean difference test was conducted by Fisher's least significant difference (LSD) test at the level of *P* <0.05. In the Figures, the data are presented as means (*n* = 3) ± SD.

To comprehensively study the responses among the metabolites (i.e., the reactive oxygen species, ascorbic acid/glutathione/nicotinamide adenine dinucleotide phosphate, and hormone levels) in the roots and shoots of cadmium-exposed *B. napus* plants treated with either 0, 50, or 100 mg kg^−1^ glutathione, we performed a normalized heatmap matrix system analysis with hierarchical clustering, a correlation coefficient analysis, and a principal component analysis (PCA). The heatmap generation, cluster analysis, and PCA were all performed in MetaboAnalyst 4.0 (www.metaboanalyst.ca; Chong et al., 2018). The data were then normalized with sum and mean-centered and divided by the standard deviation of each variable, in terms of auto-scaling. In particular, the normalized hierarchical clustering heatmap matrix was generated using a Euclidean distance measurement and Ward clustering algorithm. The plots of the PCA scores were displayed with 95% confidence regions. The correlation coefficient heatmaps were generated by Pearson correlation coefficient (*r*) distance measurements.

## Results

### GSH-Induced Cd Detoxification Improves Shoot Growth in Cd-Stressed Oilseed Rape Plants

The analyses of the *B. napus* plants at the five-leaf stage grown in the presence of 10 μM cadmium for 10 days with or without foliar glutathione treatments (50 and 100 mg kg^−1^) revealed that the plants that were subjected to cadmium alone exhibited growth inhibition (Table 1). Particularly, the Cd-stressed seedlings exhibited reductions in shoot FW and DW, but there were no changes in root DW or shoot WC. Furthermore, plant growth characteristics were not significantly different between seedlings treated with GSH alone (no Cd) and control seedlings (no Cd or GSH treatment). Nonetheless, the exogenous application of GSH to Cd-exposed *B. napus* plants considerably mitigated the adverse influences of Cd on shoot FW and DW, with the exceptions of root DW and shoot WC (Table 1).

**Table 1:** Shoot fresh weight (FW), shoot dry weight (DW), root DW, and shoot water content (WC) in oilseed rape plants grown under control [no cadmium (Cd) or glutathione (GSH)] or 0 and 10 μM Cd stresses in with/without glutathione (GSH; 0, 50, and 100 mg kg^−1^) conditions.

**Treatment**	**Shoot FW (g)**	**Shoot DW (g)**	**Root DW (g)**	**Shoot WC (%)**
Control	64.5 ± 0.91 a	4.73 ± 0.20 ab	0.33 ± 0.03 a	92.7 ± 0.36 a
0 μM Cd + 50 mg kg^–1^ GSH	64.7 ± 1.07 a	4.81 ± 0.10 a	0.34 ± 0.04 a	92.5 ± 0.02 a
0 μM Cd + 100 mg kg^–1^ GSH	64.3 ± 0.97 a	4.82 ± 0.07 a	0.32 ± 0.03 a	92.6 ± 0.17 a
10 μM Cd + 0 mg kg^–1^ GSH	54.7 ± 2.20 c	3.91 ± 0.18 c	0.30 ± 0.05 a	92.6 ± 0.65 a
10 μM Cd + 50 mg kg^–1^ GSH	61.2 ± 0.75 b	4.49 ± 0.18 b	0.32 ± 0.03 a	92.6 ± 0.24 a
10 μM Cd + 100 mg kg^–1^ GSH	63.3 ± 1.87 ab	4.61 ± 0.07 ab	0.31 ± 0.04 a	92.7 ± 0.33 a

*Plant growth characteristics were measured 10 days after treatment*.

*Means with the same lower case letter in each column are not significantly different among treatments at the P < 0.05 level based on Fisher's least significant difference (LSD) test*.

### GSH Induces Cd Uptake and Accumulation

**Figure 1:**
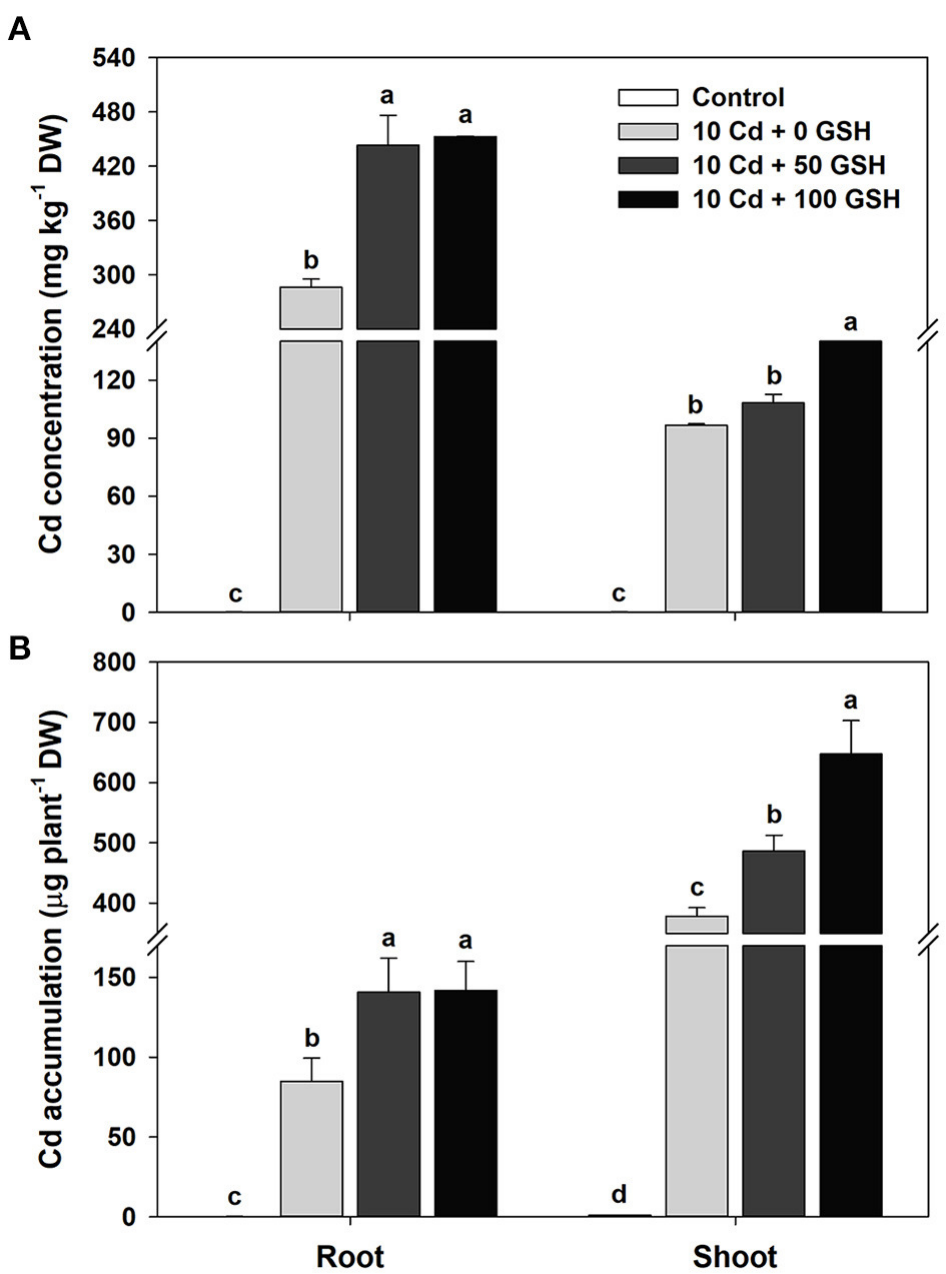
Cadmium (Cd) **(A)** concentration and **(B)** accumulation in the roots and shoots of oilseed rape plants grown under control [no Cd or glutathione (GSH)] or 10μM Cd stress conditions sprayed with/without GSH (0, 50, and 100mg kg^−1^). The concentration and accumulation of Cd were measured 10 days after treatment. Means denoted by the same letter are not significantly different at the *P* < 0.05 level according to Fisher's least significant difference (LSD) test.

The cadmium-treated plants subjected to glutathione treatments exhibited significantly enhanced cadmium uptake and accumulation, while the cadmium concentrations in the organs of seedlings treated with glutathione (50 and 100 mg kg^−1^) were higher than those of cadmium treatment alone (Figure 1A). Since the estimation of Cd depositions in the shoots and roots were based on biomass, Cd accumulation results exhibited trends similar to that of Cd concentration. Specifically, the Cd accumulations in the 50 and 100 mg kg^−1^ GSH treatments were 1.66- and 1.67-fold higher in the roots and 1.29- and 1.71-fold higher in the shoots than in the treatment with Cd alone, respectively (Figure 1B).

### Responses of GSH-Mediated ROS and MDA

**Figure 2:**
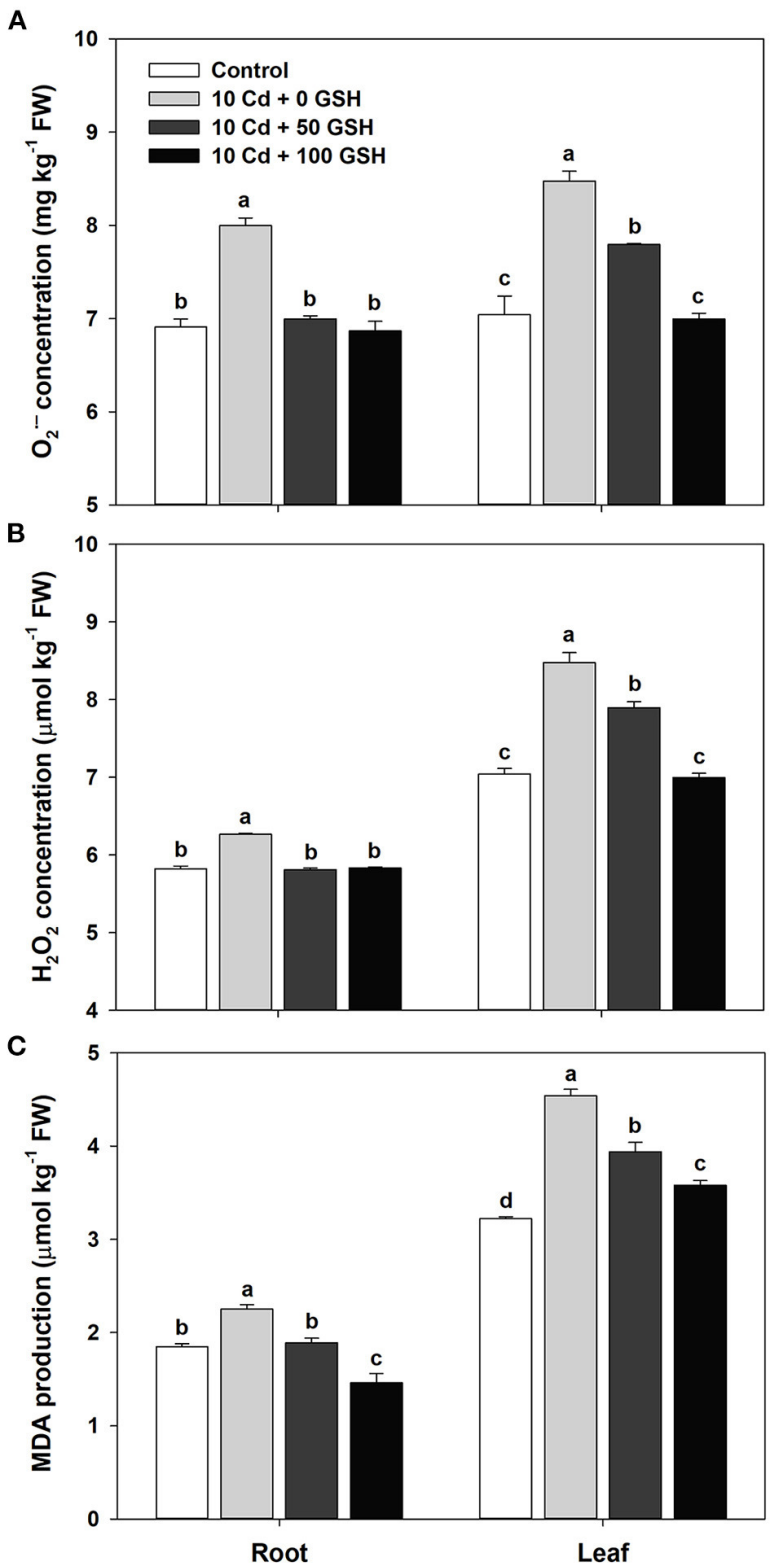
**(A)** Superoxide (${\text{O}}_{2}^{•-}$), **(B)** hydrogen peroxide (H_2_O_2_), and **(C)** malondialdehyde production (MDA) **(C)** in the roots and leaves of oilseed rape plants grown under control (no Cd or GSH) or 10μM Cd stress conditions and sprayed with/without GSH (0, 50, and 100mg kg^−1^). Reactive oxygen species (ROS) and MDA concentration were measured 10 days after treatment. Means denoted by the same letter are not significantly different at the *P* < 0.05 level according to Fisher's least significant difference (LSD) test.

To evaluate alterations in biochemical responses to cadmium toxicity in *B. napus* plants, we analyzed the ${\text{O}}_{2}^{•-}$, H_2_O_2_, and MDA concentrations in plants 10 days after the initiation of their Cd treatment. The ${\text{O}}_{2}^{•-}$ concentrations of the roots and leaves in the Cd-treated plants were 1.16- and 1.2-fold greater, respectively, than those in the corresponding organs of untreated controls (Figure 2A). Changes in the root and leaf H_2_O_2_ concentrations in response to Cd were similar to those of ${\text{O}}_{2}^{•-}$ in that that they were 1.08- and 1.13-fold greater in the roots and leaves, respectively, of the Cd-exposed plants than those in the untreated negative controls (Figure 2B). In the roots and leaves of the Cd-treated plants, the MDA concentrations were 1.22- and 1.41-fold greater, respectively, than the MDA in the corresponding organs of the untreated control plants (Figure 2C). However, in both organs of the oilseed rape plants, the exogenous treatment of Cd-stressed plants with GSH at both concentrations significantly reduced the concentration of ${\text{O}}_{2}^{•-}$, H_2_O_2_, and MDA relative to the levels in the plants treated with Cd alone (Figures 2A–C).

### GSH-Mediated Modulation of the AsA-GSH-NADPH Cycle Mitigates Cellular Redox Imbalance in Cd-Stressed Oilseed Rape Plants

**Figure 3:**
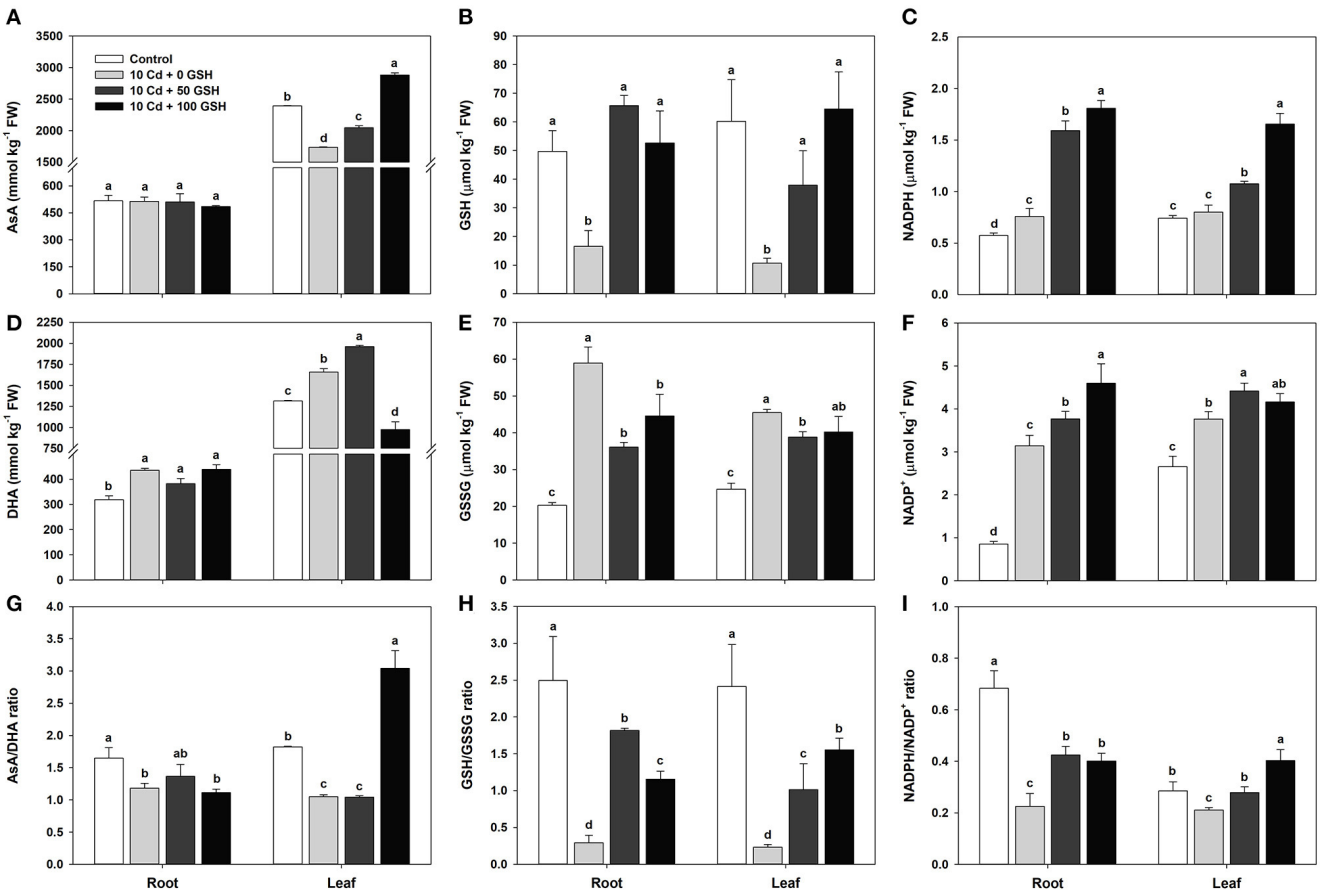
Ascorbate (AsA, reduced form)/GSH (reduced form)/nicotinamide adenine dinucleotide phosphate (NADPH, reduced form); dehydroascorbate (DHA, oxidized form)/GSH disulfide (GSSG, oxidized form)/nicotinamide adenine dinucleotide (NADP^+^, oxidized form) concentration; the AsA/GSH/NADPH redox ratio in the roots and leaves of oilseed rape plants grown under control (no Cd or GSH) or 10μM Cd stress conditions sprayed with/without GSH (0, 50, and 100mg kg^−1^). Measurements were made 10 days after treatment. Concentrations of **(A–C)** AsA, GSH, and NADPH; concentrations of **(D–F)** DHA, GSSG, and NADP^+^; **(G–I)** AsA, GSH, and NADPH redox ratios. Data are presented as mean ± SD (*n* = 3). Means denoted by the same letter are not significantly different at the *P* < 0.05 level according to Fisher's least significant difference (LSD) test.

The concentrations of ascorbic acid, glutathione, and nicotinamide adenine dinucleotide phosphate and redox status of the *B. napus* plants exposed to cadmium stress and 0, 50, or 100mg kg^−1^ glutathione were assessed after 10 days. For the plants treated with Cd only, the AsA concentration of the roots did not change, but foliar AsA concentration decreased relative to levels in the untreated control plants (Figure 3A). In contrast, the oxidized form of AsA (DHA concentration) significantly increased in the roots and leaves following Cd treatment alone compared with that in the untreated control plants (Figure 3D). The AsA-redox ratio was also significantly reduced in both organs of the Cd-treated plants compared with those in the untreated plants (Figure 3G).

However, when the cadmium-stressed plants simultaneously received foliar applications of glutathione, their ascorbic acid concentration did not change in the roots but significantly increased in the leaves compared with seedlings that received Cd alone (Figure 3A). Furthermore, the DHA concentration in the roots was not significantly different between Cd alone and the Cd + GSH treatments (50 and 100mg kg^−1^). Meanwhile, the DHA concentration of the leaves increased with 50 mg kg^−1^ GSH treatment but greatly decreased with the application of 100 mg kg^−1^ GSH compared with that of the cadmium-stressed plants without glutathione (Figure 3D). In summary, the AsA-redox ratio in the roots was not significantly different, whereas ghe high AsA-redox ratio in the leaves was maintained, owing to the increased AsA level and reduced DHA level following GSH application as compared with levels in the Cd treatment alone (Figure 3G).

Glutathione concentration was significantly lower in the plants treated with Cd only relative to untreated control plants, but not in the glutathione-treated plants (Figure 3B). In contrast to the decrease in GSH concentration in the Cd-only exposed plants, GSSG (the oxidized form of GSH) levels were considerably higher in the Cd treatment than in the untreated controls (Figure 3E). Moreover, the GSH-redox ratio in the Cd-only treatment was reduced in both plant organs compared with the untreated control (Figure 3H).

The glutathione concentration in the plants that underwent both glutathione treatments (50 and 100 mg kg^−1^) increased markedly in both the roots and leaves compared with those of plants exposed to cadmium alone (Figure 3B). The GSSG concentration in both organs was significantly lower in the 50-mg kg^−1^ GSH treatment than in the Cd-alone treatment. However, the GSSG concentration in the 100-mg kg^−1^ GSH-treated plants was significantly lower in the roots, but no change was observed in the leaves compared with that in the Cd treatment alone (Figure 3E). Noticeably, the GSH treatments significantly increased the GSH-redox ratio in both organs when compared with those of the plants that underwent the Cd-alone treatment (Figure 3H).

The concentration of nicotinamide adenine dinucleotide phosphate in the cadmium-stressed plants slightly increased in the roots, but no significant difference was observed in the leaves relative to the untreated control plants (Figure 3C). The concentration of NADP^+^ was significantly enhanced in both organs (Figure 3F). The NADPH-redox ratio in the Cd-exposed plants was significantly decreased in roots and leaves compared with the untreated plants (Figure 3I). Notably, however, NADPH levels in the Cd-stressed *B. napus* seedlings with GSH applications (50 and 100 mg kg^−1^) increased significantly in the roots and leaves when compared with those in the Cd treatment alone (Figure 3C). The NADP^+^ level was significantly higher in the roots of plants treated with foliar GSH than in the roots of plants treated only with Cd. Similarly, the NADP^+^ level increased significantly in the leaves of the plants treated with 50 mg kg^−1^ GSH, but not 100 mg kg^−1^ GSH, compared with levels in the leaves of plants that received only the Cd treatment (Figure 3F). Nevertheless, the NADPH-redox ratios in both organs were significantly increased by exogenous GSH treatments when compared with that of Cd treatment alone (Figure 3I).

### GSH-Mediated Modulation of Enzymatic Antioxidant Defense Systems Reestablishes Antioxidant and Redox Enzyme Homeostasis in Cd-Stressed Oilseed Rape Plants

**Figure 4:**
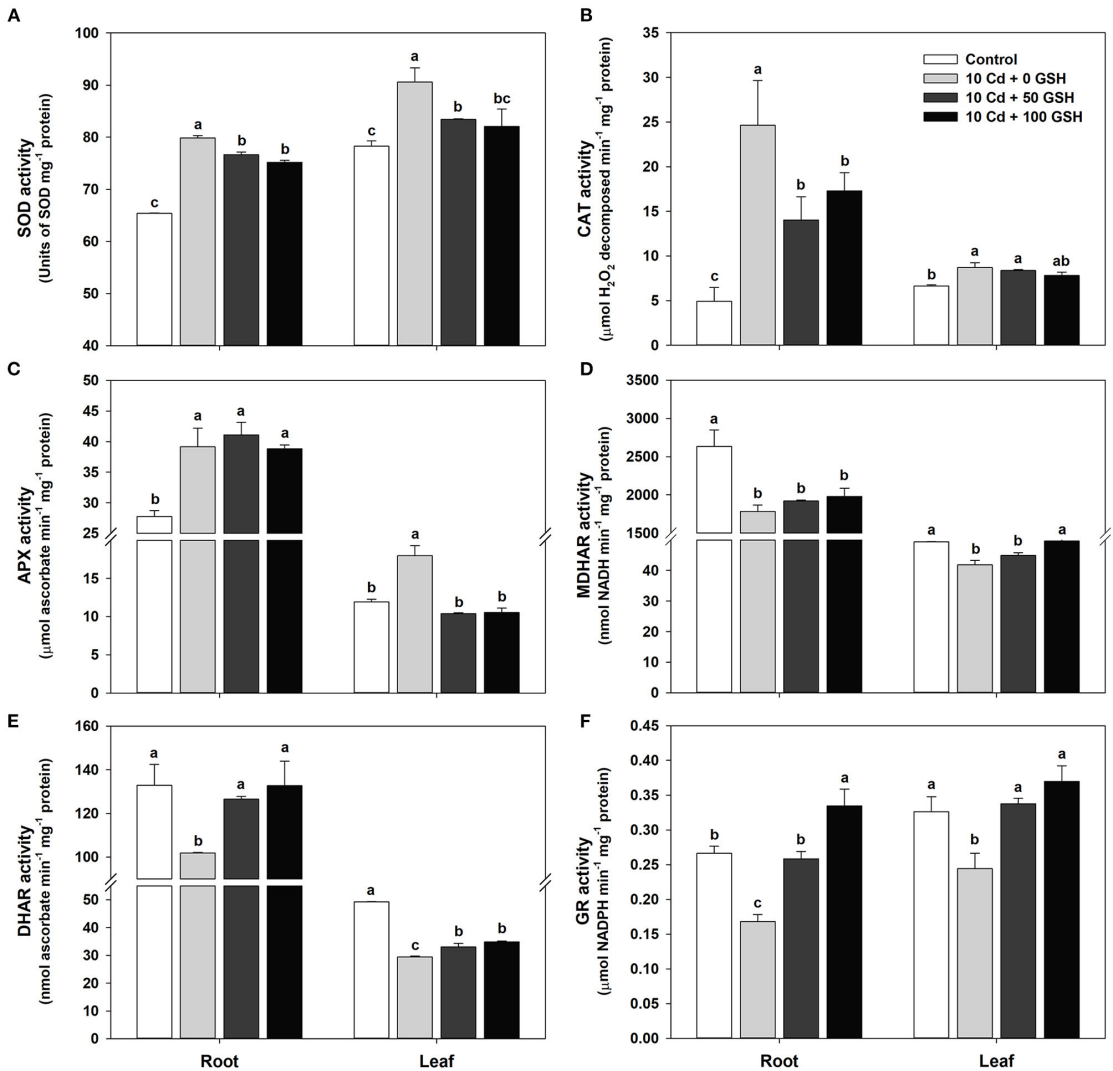
Activities of antioxidant enzymes in the roots and leaves of oilseed rape plants grown under control (no Cd or GSH) or 10μM Cd stress conditions sprayed with/without GSH (0, 50, and 100mg kg^−1^). **(A)** Superoxide dismutase (SOD), **(B)** catalase (CAT), **(C)** ascorbate peroxidase (APX), **(D)** monodehydroascorbate reductase (MDHAR), **(E)** dehydroascorbate reductase (DHAR), and **(F)** GSH reductase (GR) activities. The activity of each antioxidant enzyme was measured 10 days after treatment. Data are presented as mean ± SD (*n* = 3). Means denoted by the same letter are not significantly different at the *P* < 0.05 level according to Fisher's least significant difference (LSD) test.

Applications of glutathione to cadmium-stressed *B. napus* plants have been observed to curtail cadmium-induced oxidative stress (Figure 2). In this section, the enzyme activities of SOD, CAT, APX, MDHAR, DHAR, and GR were measured to help elucidate the effect of exogenous GSH application in Cd-treated plants on antioxidant scavenging defense systems. Particularly, that SOD, CAT, and APX activities in plants treated with Cd only increased significantly in the roots and leaves of oilseed rape plants (Figures 4A–C), whereas MDHAR, DHAR, and GR activities decreased significantly in both organs, relative to the activities in the untreated plants (Figures 4D–F). Furthermore, GSH application to the Cd-treated seedlings significantly decreased the SOD activity in both organs compared with that in plants treated only with Cd (Figure 4A). Similarly, CAT activity was significantly reduced in the roots of plants treated with GSH, whereas no significant difference was found in the leaves compared with the Cd-only treated plants (Figure 4B). No significant difference was observed in APX activity in the roots of the GSH-treated and Cd-only treated plants; however, APX activity decreased significantly in the leaves of the GSH-treated plants (Figure 4C). Additionally, MDHAR activity in the GSH-treated plants did not change in the roots or in the leaves treated with 50 mg kg^−1^ GSH, whereas it significantly increased with the application of 100 mg kg^−1^ of GSH compared with the Cd treatment alone (Figure 4D). The activities of DHAR and GR in the GSH-treated plants were considerably higher in both organs than in the Cd-stressed plants alone (Figures 4E,F).

### GSH-Mediated Modulation of Stress-Associated Hormones Maintains Cellular Hormone Balance in Cd-Stressed Oilseed Rape Plants

**Figure 5:**
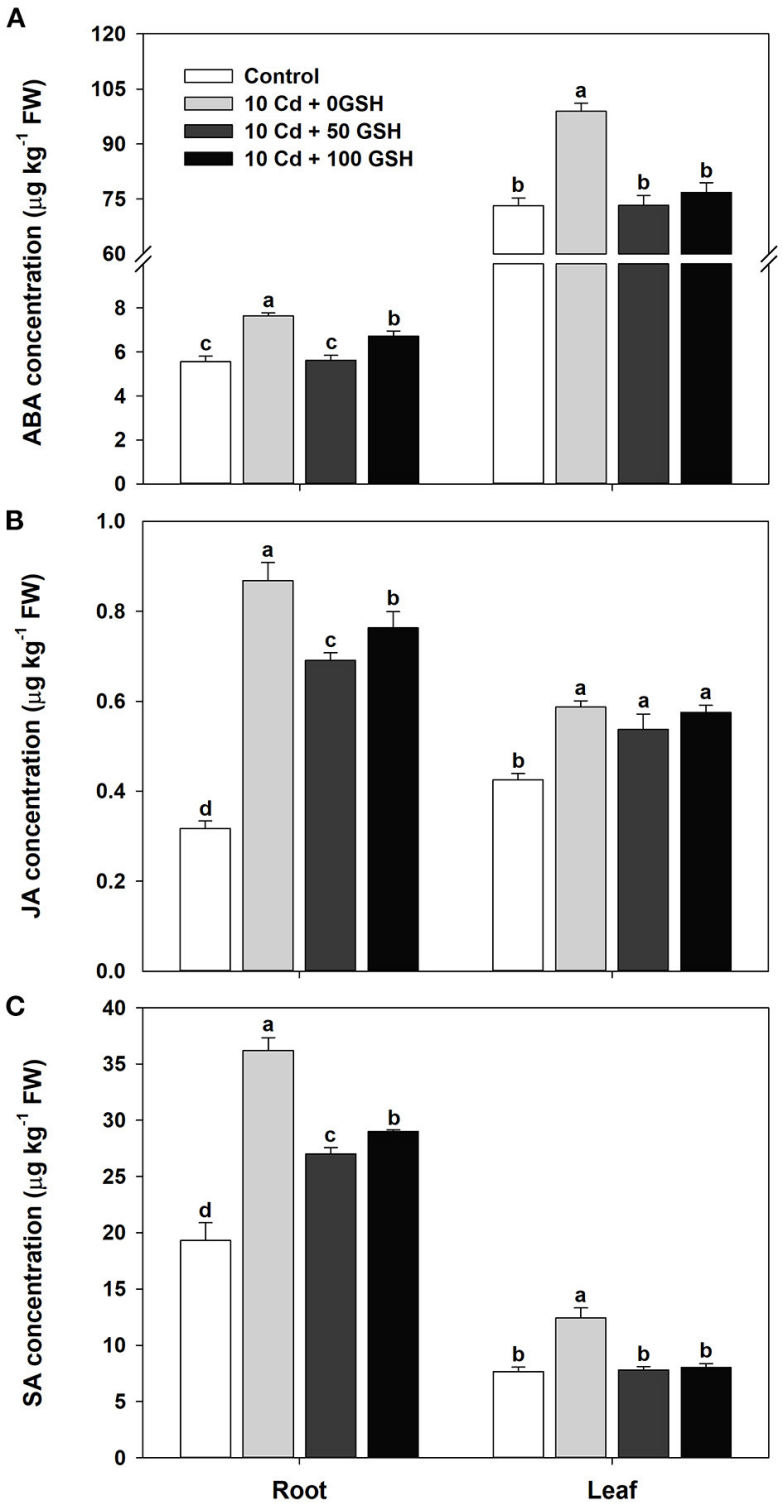
Stress-associated hormone concentrations in the roots and leaves of oilseed rape plants grown under control (no Cd or GSH) or 10μM Cd stress conditions sprayed with/without GSH (0, 50, and 100mg kg^−1^). **(A)** Abscisic acid (ABA), **(B)** jasmonic acid (JA), and **(C)** salicylic acid (SA) **(C)** concentrations. The concentration of each hormone was measured 10 days after treatment. Data are presented as mean ± SD (*n* = 3). Means denoted by the same letter are not significantly different at the *P* < 0.05 level according to Fisher's least significant difference (LSD) test.

The levels of the stress-inducible hormones abscisic acid, jasmonic acid, and salicylic acid were determined to assess the influence of glutathione on hormone homeostasis in cadmium-exposed plants. In general, the concentrations of these hormones in the roots and leaves were significantly increased in the Cd-stressed plants compared with the untreated plants (Figures 5A–C). The exogenous treatment of Cd-treated plants with 50 and 100mg kg^−1^ of GSH significantly lowered ABA and SA concentrations in both organs compared with the plants that only received the Cd treatment (Figures 5A,C). Similarly, the concentration of JA was significantly reduced in the roots by the GSH treatment, but no change was observed in the leaves compared with those of the Cd treatment alone (Figure 5B).

### Comparative Correlations of Observed ROS, Redox, and Hormonal Responses to Cd Stress in Oilseed Rape Plants

**Figure 6:**
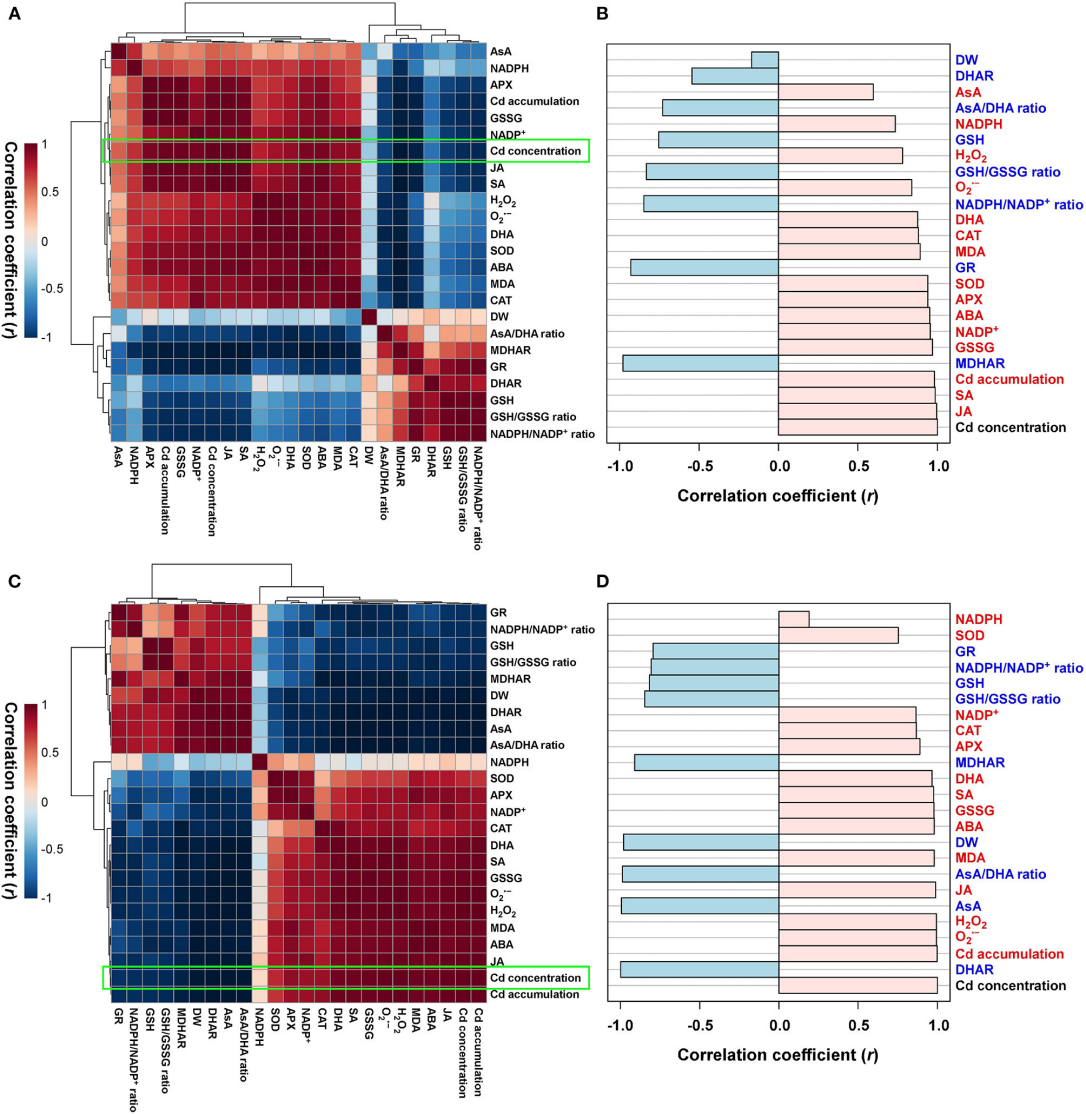
Heatmap analysis of Pearson's correlation coefficient (*r*) for the targeted metabolites of ROS, AsA/GSH/NADPH redox status, and stress-associated hormone status in the **(A,B)** roots and **(C,D)** shoots of Cd-stressed seedlings.

For the cadmium-stressed plants, the normalized heatmap matrix analyses of both the roots and shoots mainly classified the observed responses to cadmium into two groups, which both revealed positive correlations. First, positive correlations were observed among Cd translocation, ROS, antioxidant defense system, and stress-hormone-associated components. Second, positive correlations were identified among AsA/GSH/NADPH-redox constituents (Figures 6A,C). A comparative analysis of the factors that correlated with Cd concentration (presented by the bright green boxes in Figures 6A,C) indicated that positive correlations existed between the concentration of Cd in the roots and the concentrations of stress hormones (JA, SA, and ABA), Cd accumulation, oxidized forms of antioxidants (GSSG, NADP^+^, and DHA), the activity of antioxidant enzymes (APX, SOD, and CAT), the concentration of oxidative stress components (MDA and ${\text{O}}_{2}^{•-}$, and H_2_O_2_), NADPH, and AsA concentration. In this study, it was also observed that the concentration of Cd in the roots negatively correlated with the activities of antioxidant enzymes (MDHAR, GR, and DHAR), redox ratios (NADPH/NADP^+^, GSH/GSSG, and AsA/DHA), GSH concentration, and DW (Figure 6B). Conversely, positive correlations were observed to exist between the concentration of Cd in the shoots and Cd accumulation, oxidative stress components (${\text{O}}_{2}^{•-}$, H_2_O_2_, and MDA), stress hormones (JA, ABA, and SA), oxidized forms of antioxidants (GSSG, DHA, and NADP^+^), antioxidant enzymes (APX, CAT, and SOD), and NADPH concentration; whereas, negative correlations were observed with the activities of antioxidant enzymes (DHAR, MDHAR, and GR), the concentrations of antioxidants in their reduced forms (AsA and GSH), redox ratios (AsA/DHA, GSH/GSSG, and NADP/NADP^+^ ratios), and DW (Figure 6D).

### Normalized Heatmap Responses to Cd Stress With or Without GSH Applications in Oilseed Rape Plants

**Figure 7:**
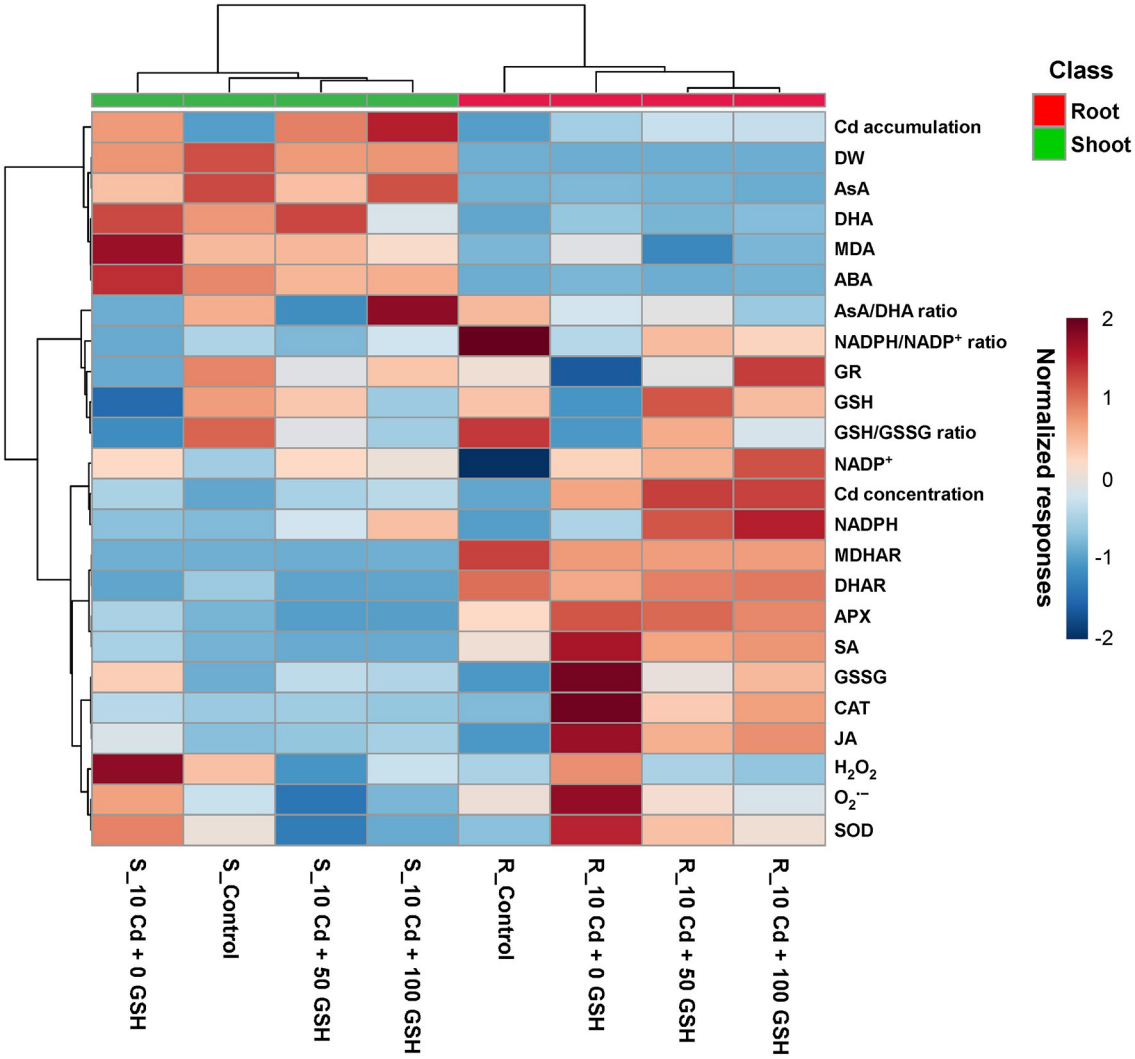
Normalized heatmap matrix of observed responses in ROS levels, AsA/GSH/NADPH redox status, and stress-associated hormone status in the roots and shoots of Cd-stressed plants treated with different GSH levels. R, root; S, shoot.

Oilseed rape plant responses to glutathione treatments under cadmium stress conditions were evaluated using a normalized heatmap matrix with hierarchical clustering (Figure 7). The GSH and Cd treatments caused differential responses between the roots and the shoots. Furthermore, the GSH treatments clustered more closely with the untreated control treatment than the Cd-only treatment, irrespective of the roots and shoots. Meanwhile, based on the relative responses among the response variables, the sole Cd treatment was the most distantly clustered from the other three treatments, regardless of the type of organ.

In particular, the H_2_O_2_, ${\text{O}}_{2}^{•-}$, and superoxide dismutase in both organs had relatively higher normalized responses in the Cd-only treatment than in the other treatments, regardless of glutathione application rate. Specifically, Cd accumulation, DW, AsA, DHA, MDA, ABA, and AsA/DHA ratio exhibited relatively higher normalized responses in the shoots than in the roots. However, the normalized responses in the NADPH/NADP^+^ and GSH/GSSG ratios, Cd concentration, and GR, GSH, NADP^+^, NADPH, MDHAR, DHAR, APX, SA, GSSG, CAT, and JA were relatively higher in the roots than in the shoots (Figure 7).

### Principal Component Analysis of the Targeted Metabolites of ROS, AsA/GSH/NADPH-Redox, and Stress-Associated Hormone Status of Cd-Stressed Oilseed Rape Plants With or Without GSH Application

**Figure 8:**
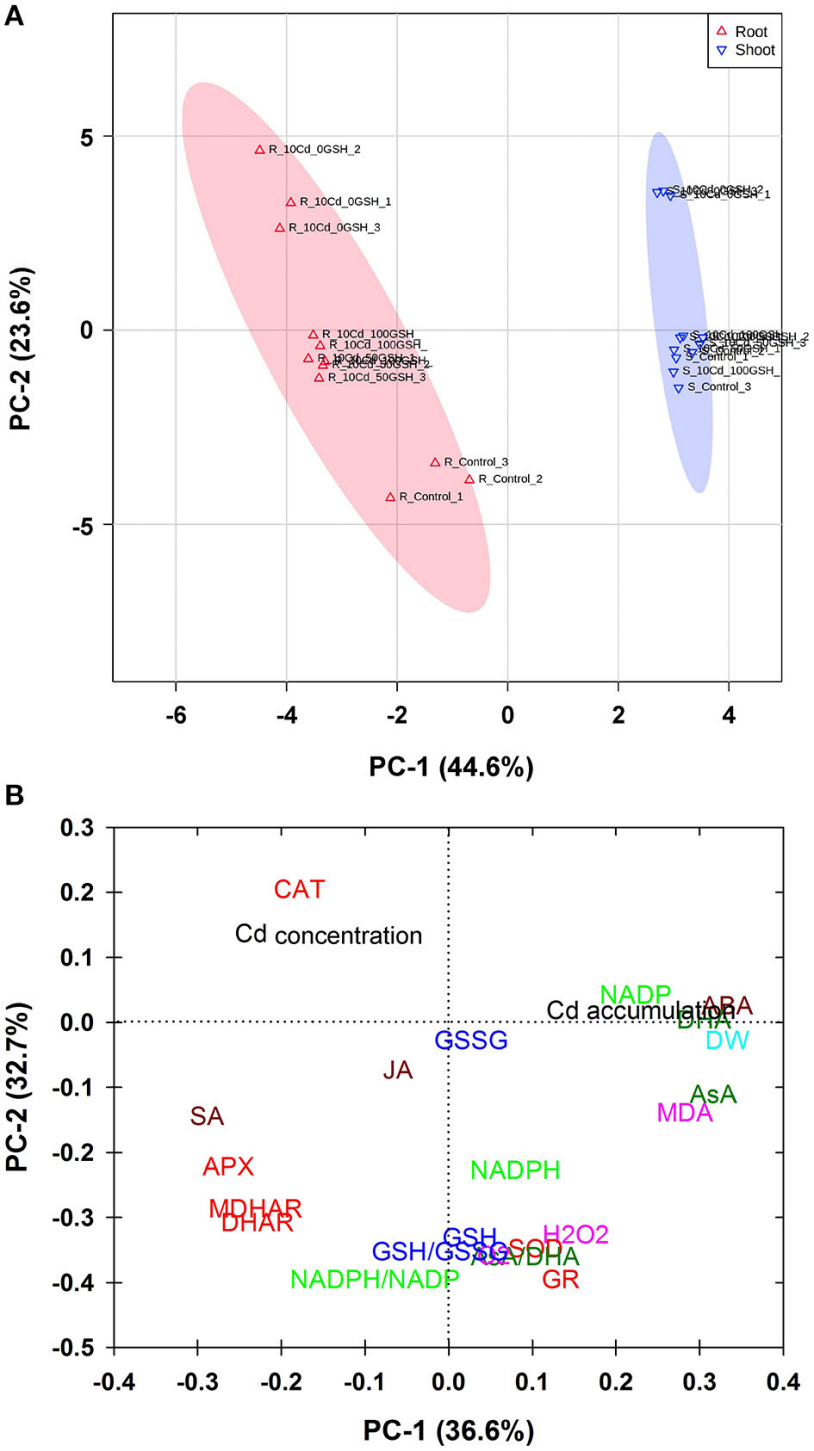
**(A)** Plot of principal component analysis (PCA) scores of untreated controls (no Cd or GSH) or Cd-stressed plants receiving 0, 50, or 100mg kg^−1^ GSH and **(B)** loading plot of ROS, AsA/GSH/NADPH redox status, and stress-associated hormone status in the roots and shoots of Cd-stressed plants treated with different GSH concentrations. R, root; S, shoot.

A principal component analysis score plot was adopted for the estimation of the comprehensive responses of oilseed rape plants to glutathione treatments under Cd toxicity conditions (Figure 8A). The PC-1 and PC-2 values accounted for 44.6 and 23.6% of the total x- and y-variances, respectively. There were distinct, separated clustering patterns detected between the roots and shoots of the oilseed rape plants. In both organs, the Cd-alone treatment was distinctively segregated from the untreated control and the treatments wherein the plants received both Cd and foliar GSH. However, the results showed that both the roots and the shoots exhibited similar patterns in their physiological and biochemical responses, and these responses were proportional to the levels of GSH applied. The responses in both organs became increasingly similar to those of the untreated controls with an increase in the amount of GSH applied. These results imply that Cd-toxicity mitigation partially depends on the concentration of GSH.

A principal component analysis loading plot was used to assess associations among the response variables (Figure 8B). There were four major associations, which were as follows: (a) CAT and Cd concentrations were closely linked with each other; (b) SA, APX, MDHAR, and DHAR were also tightly associated with each other; (c) NADPH/NADP^+^, GSH/GSSG, and AsA/DHA ratios, and the GSH, SOD, GR, H_2_O_2_, ${\text{O}}_{2}^{•\text{–}}$, and NADPH levels were closely related to one another; (d) NADP^+^, Cd accumulation, ABA, DHA, DW, AsA, and MDA were closely associated with each other.

## Discussion

Cadmium is one of the most toxic heavy metals and is highly mobile in both soils and plants (Hussain et al., 2021). Owing to its inhibition of multiple physiological, biochemical, and metabolic processes, Cd is deleterious to normal plant growth and development (Jan et al., 2015). Therefore, much research effort has been devoted to finding means to alleviate Cd phytotoxicity. Recently, numerous studies have demonstrated that the application of AsA (Zhang et al., 2019; Jung et al., 2020), GSH (Ding et al., 2017; Jung et al., 2019), H_2_O_2_ (Asgher et al., 2020), hydrogen sulfide (Corpas and Palma, 2020; Luo et al., 2020), nitric oxide (Singh A. P. et al., 2016), sulfur (Tian et al., 2017; Lu et al., 2019), ABA (Shi et al., 2019; Zhu et al., 2020), JA (Bali et al., 2019), SA (Kovács et al., 2014; Guo et al., 2016), or polyamines (Pál et al., 2017) can help mitigate plant stress from several heavy metals.

In this study, we demonstrated that the application of glutathione to cadmium-stressed *B. napus* plants reduced the growth inhibition caused by Cd toxicity in terms of shoot fresh weight and dry weight, whereas root dry weight and shoot water content were not affected by cadmium exposure (Table 1). Nevertheless, Cd concentration and accumulation were significantly higher in the roots and shoots of Cd-stressed *B. napus* plants following GSH application than those in the plants treated with Cd alone (Figures 1A,B), which is consistent with the results of previous reports showing that exogenous GSH application increases Cd accumulation and tolerance in barley (Chen et al., 2010) and rice (Cai et al., 2010) seedlings. The results of this study showed that GSH application induced the uptake and accumulation of Cd in the roots and shoots of *B. napus*, while it alleviated the overall growth inhibition caused by Cd toxicity, subsequently indicating GSH-induced improvement in Cd tolerance. Furthermore, GSH functions as a Cd chelator, leading to the formation of Cd-GSH complexes that promote Cd uptake through the roots and enhance Cd accumulation in the vacuole (Seth et al., 2012; Anjum et al., 2014; Ding et al., 2017; Bellini et al., 2021). In addition, phytochelatins (PCs), which are GSH-derived polypeptides produced by PC synthase, chelate with Cd to form PCs-Cd complexes in the cytosol, which are then transported and sequestered into the vacuoles through ATP-binding cassette (ABC) transporters, thereby inducing the detoxification of Cd in plant cells (Ueno et al., 2010; Yadav, 2010; Park et al., 2012; Zagorchev et al., 2013; Semida et al., 2018; Zhang et al., 2018). In *Arabidopsis thaliana*, the overexpression of PC synthase 1 (PCS1) enhanced Cd tolerance and accumulation because of the increase in GSH and PC contents (Guo et al., 2008). Therefore, the chelation of GSH or PCs with Cd might be an important component involved in Cd detoxification and Cd accumulation in *B. napus* plants.

The uptake and accumulation of cadmium in plants generate reactive oxygen species (${\text{O}}_{2}^{•-}$, H_2_O_2_, and OH^•^) by replacing the cofactors of essential-metal ions in metalloproteins *via* the Fenton reaction (Loix et al., 2017), which, in turn, damages cell membranes through lipid peroxidation and inhibits the biosynthesis of cellular biomolecules required for plant life (Yan et al., 2015). Various studies have demonstrated that Cd induces ROS overproduction and growth inhibition in numerous plants (Loix et al., 2017). This study also verified that Cd toxicity caused oxidative stress by the excessive production of ${\text{O}}_{2}^{•-}$ (Figure 2A) and H_2_O_2_ (Figure 2B) in the roots and shoots of *B. napus* plants. As the levels of the ROS increased, a proportional increase in MDA production, implying the existence of lipid peroxidation under oxidative stress conditions, was observed in the Cd-stressed plants (Figure 2C). However, exogenous GSH application to Cd-stressed *B. napus* plants significantly decreased ROS and MDA levels in the roots and shoots (Figure 2), indicating that GSH plays an important role in the mitigation of oxidative stress produced by Cd toxicity. Jung et al. (2019) recently reported that the exogenous application of GSH to As-stressed rice plants restricted the generation of oxidative stress and lipid peroxidation, as evidenced by the decreased MDA production of the tested plants. In addition, exogenous PCs synthesized from GSH as mentioned above increase the scavenging activity of H_2_O_2_ and ${\text{O}}_{2}^{•-}$ induced by Zn stress (Tsuji et al., 2002), indicating that exogenous GSH-induced PCs may be directly involved in the removal of ROS in Cd-exposed plants. This study provides evidence that the GSH-mediated decline of oxidative stress in Cd-stressed *B. napus* plants occurs through either its direct or indirect antioxidant effects, thereby either reducing MDA production or altering the antioxidant scavenging system in a manner that lowers the level of ROS (Figure 2).

Along with ascorbic acid and nicotinamide adenine dinucleotide phosphate, glutathione is part of the plant non-enzymatic defense system against oxidative stress that involves antioxidants and redox metabolites. In response to Cd toxicity, GSH primarily acts as an antioxidant that is associated with detoxifying ROS, but it also plays a critical secondary role as a redox buffer (Zhang et al., 2013). In this study, Cd treatment significantly reduced the GSH levels in both organs of the *B. napus* plants, but only reduced the AsA level in leaves (Figures 3A,B). In contrast, the level of NADPH in the Cd-exposed roots slightly increased, while there was no change in the Cd-stressed leaves (Figure 3C). Interestingly, there were increases in the concentrations of DHA, GSSG, and NADP^+^, whereas the AsA/DHA, GSH/GSSG, and NADPH/NADP^+^ ratios concomitantly decreased (Figures 3D–I). Moreover, Cd-induced oxidative stress in the *B. napus* plants was associated with the redox status of AsA, GSH, and NADPH (Figures 2, 3).

However, the exogenous application of glutathione improved the levels of ascorbic acid, glutathione, and nicotinamide adenine dinucleotide phosphate and the ratios of AsA/DHA, GSH/GSSG, and NADPH/NADP^+^ (Figure 3), indicating that redox cycle activities were strengthened and that these suppressed the ROS generated by Cd toxicity. Consistent with these results, Jung et al. (2019) and Chen et al. (2010) opined that the exogenous application of GSH reduced As- and Cd-caused oxidative stresses, decreased lipid peroxidation, increased GSH levels, and maintained cellular redox balances in rice (As toxicity) and barley (Cd toxicity). Therefore, these detoxification systems play a crucial role in the maintenance of cellular oxidation/reduction status and membrane functional and structural integrity (Jung et al., 2019).

In addition to the non-enzymatic antioxidants and redox metabolites, plants have a second pathway for mitigating oxidative stress involving antioxidant and redox enzymes. Among these enzymes, SOD induces the dismutation of ${\text{O}}_{2}^{•-}$ to H_2_O_2_, whereas CAT and APX convert H_2_O_2_ into H_2_O and O_2_. The activities of SOD, CAT, and APX scavenge ROS, which otherwise cause oxidative damage, and then convert them to harmless H_2_O and O_2_ (Shekhawat et al., 2010). In this study, high activities of SOD, CAT, and APX were observed under conditions of Cd stress, as ROS increased with Cd uptake and accumulation (Figures 1A,B); however, the activities of these enzymes were significantly reduced by GSH application (Figures 4A–C). At the same time, the effects of GSH application on enzyme activity were correlated with decreased ROS levels in the Cd-exposed seedlings, indicating that the damage associated with oxidative stress was minimized even as SOD, CAT, and APX activities declined (Figure 2).

The seemingly paradoxical concomitant decreases in both reactive oxygen species and the activities of the redox enzymes superoxide dismutase, catalase, and ascorbate peroxidase in response to exogenous GSH is resolved once the responses of MDHAR, DHAR, and GR are considered. In contrast to the activities of the redox enzymes, the activities of the core enzymes, MDHAR, DHAR, and GR, which are involved in the AsA/GSH/NADPH-redox cycle, markedly decreased under Cd stress conditions in the roots and leaves of *B. napus* plants. However, exogenous GSH application considerably increased the MDHAR, DHAR, and GR activities in both organs, with the exception of the MDHAR activity in roots, compared with the Cd treatment alone (Figures 4D–F). The alterations in antioxidant enzyme activities occurring under Cd toxicity conditions were also reversed by GSH application; their activities recovered to levels close to those in the untreated control plants (Figures 7, 8). The results then showed that exogenous GSH application improved plant defenses against Cd-induced oxidative stress by increasing core antioxidant enzyme activities, which partially underlies the concomitant observed reductions in the levels of ${\text{O}}_{2}^{•-}$, H_2_O_2_, and MDA in the *B. napus* plants. Thus, these findings suggest that GSH not only directly reduces ROS by acting as an antioxidative ROS scavenger, but it also indirectly mitigates Cd-induced ROS overproduction through the modulation of antioxidant enzyme activities. Therefore, the redox balance of the plant is maintained in response to endogenous GSH levels.

Several studies on the relationship between heavy metals and phytohormones have reported that abscisic acid (Shi et al., 2019; Zhu et al., 2020), jasmonic acid (Bali et al., 2019), and salicylic acid (Kaya et al., 2020) curtail the oxidative stress caused by heavy metals in various plants. Plant hormone balance is, therefore, likely to be informative for understanding plant resistance to Cd toxicity. In this study, ABA, JA, and SA levels significantly increased in the Cd-stressed plants in comparison with the untreated plants. However, GSH application improved the ABA, JA, and SA levels, which might decrease the oxidative stress caused by Cd through the activation of the AsA-GSH-NADPH cycle to overcome Cd toxicity (Figure 5). These results suggest that GSH can mitigate the detrimental effects of Cd on hormone homeostasis.

Finally, we evaluated the overall phytotoxicity of cadmium through the analysis of a heatmap (Figure 7) and principal component analysis score plot (Figures 8A,B) of the targeted metabolites in cadmium-stressed oilseed rape plants with or without glutathione treatment. The results indicated that the exogenous application of GSH minimized the phytotoxicity of Cd and promoted Cd accumulation in the roots and shoots of oilseed rape, with the benefits of increasing in proportion to the amount of GSH applied (Figures 7, 8).

**Figure 9:**
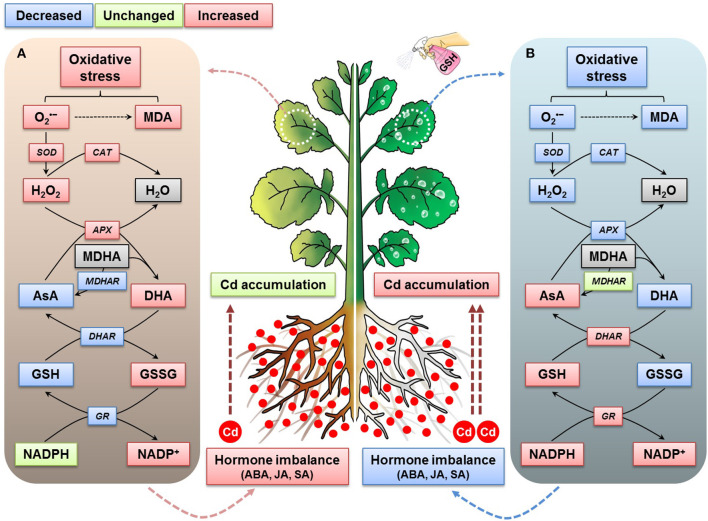
Interconnected mechanism of the GSH-mediated modulation of Cd stress responses on ROS, redox, and stress-hormone status in oilseed rape plants. **(A)** Cd-stressed plant growth status and **(B)** Cd-stressed and GSH-treated plant growth status.

We propose a comprehensive and systematic interconnected mechanism for enhancing the accumulation of cadmium in oilseed rape plants by mitigating Cd-associated oxidative stress and facilitating the homeostasis of the ascorbic acid-glutathione-nicotinamide adenine dinucleotide phosphate cycle and stress hormones. The application of GSH to Cd-stressed oilseed rape plants reduced the production of ROS and MDA significantly when compared with the effects observed in *B. napus* plants exposed to Cd treatment alone (Figure 9B). As shown here, the proposed mechanism indicates that GSH application to plants under Cd exposure conditions stimulated the AsA-GSH-NADPH cycle, enabling Cd accumulation to increase in these plants and stimulating their antioxidant-scavenging, redox-regulating, and hormone-balancing systems, which also decreased the overall Cd phytotoxicity in the oilseed rape plants (Figure 9).

## Conclusion

In this study, we demonstrated that exogenous glutathione application increased cadmium accumulation through glutathione-phytochelatin-mediated vacuolar sequestration. It also ameliorated the oxidative stress induced by Cd in oilseed rape plants by simultaneously promoting components of the antioxidant-scavenging, redox-regulating, and hormone-balancing systems. Further investigations of the mechanism of GSH transport in plants during foliar treatment and large-scale field trials are warranted to develop phytoremediation and/or sustainable crop production systems for Cd-polluted soils. Hence, the results in this study provide insights into the important functions and roles of GSH in modulating AsA-GSH-NADPH homeostasis and stress hormone balance, which contribute to the enhancement of Cd uptake, and its accumulation and resistance in oilseed rape plants. Foliar GSH application represents a feasible and practical approach for mitigating Cd stress in oilseed rape plants and increasing the efficiency of the Cd phytoextraction of Cd-contaminated soils.

## Data Availability Statement

The raw data supporting the conclusions of this article will be made available by the authors, without undue reservation.

## Author Contributions

H-iJ: conceptualization, methodology, and writing original draft. H-iJ, SJ, and B-RL: investigation. H-iJ, T-GL, JL, M-JC, E-JL, M-SK, G-BJ, SJ, and B-RL: formal analysis. H-iJ, JL, and B-RL: visualization. H-iJ, T-GL, JL, M-JC, E-JL, M-SK, G-BJ, AE, SJ, and B-RL: writing, review, and editing. All authors contributed to the article and approved the submitted version.

## Funding

This study was supported by the Cooperative Research Program for Agriculture Science and Technology Development (Project No. PJ014360), RDA, Republic of Korea.

## Conflict of Interest

The authors declare that the research was conducted in the absence of any commercial or financial relationships that could be construed as a potential conflict of interest.

## Publisher's Note

All claims expressed in this article are solely those of the authors and do not necessarily represent those of their affiliated organizations, or those of the publisher, the editors and the reviewers. Any product that may be evaluated in this article, or claim that may be made by its manufacturer, is not guaranteed or endorsed by the publisher.
